# Moloney leukemia virus 10 (MOV10) inhibits the degradation of APOBEC3G through interference with the Vif-mediated ubiquitin–proteasome pathway

**DOI:** 10.1186/s12977-017-0382-1

**Published:** 2017-12-19

**Authors:** Cancan Chen, Xiaocao Ma, Qifei Hu, Xinghua Li, Feng Huang, Junsong Zhang, Ting Pan, Jinyu Xia, Chao Liu, Hui Zhang

**Affiliations:** 1 Institute of Human Virology, Zhongshan School of Medicine, Sun Yat-sen University, Guangzhou, 510080 China; 2 Key Laboratory of Tropical Disease Control of Ministry of Education, Zhongshan School of Medicine, Sun Yat-sen University, Guangzhou, 510080 China; 3 Department of Pathology, The First Affiliated Hospital, Sun Yat-sen University, Guangzhou, 510080 China; 4 Department of Infectious Diseases, The Fifth Affiliated Hospital, Sun Yat-sen University, Zhuhai, 519000 China

**Keywords:** MOV10, A3G, Vif, HIV-1, Ubiquitin–proteasome system (UPS)

## Abstract

**Background:**

MOV10 protein has ATP-dependent 5′–3′ RNA helicase activity and belongs to the UPF1p superfamily. It can inhibit human immunodeficiency virus type 1 (HIV-1) replication at multiple stages and interact with apolipoprotein-B-mRNA-editing enzyme catalytic polypeptide-like 3G (APOBEC3G or A3G), a member of the cytidine deaminase family that exerts potent inhibitory effects against HIV-1 infection. However, HIV-1-encoded virion infectivity factor (Vif) protein specifically mediates the degradation of A3G via the ubiquitin–proteasome system (UPS).

**Results:**

We demonstrate that MOV10 counteracts Vif-mediated degradation of A3G by inhibiting the assembly of the Vif-CBF-β-Cullin 5-ElonginB-ElonginC complex. Through interference with UPS, MOV10 enhances the level of A3G in HIV-1-infected cells and virions, and synergistically inhibits the replication and infectivity of HIV-1. In addition, the DEAG-box of MOV10 is required for inhibition of Vif-mediated A3G degradation as the DEAG-box mutant significantly loses this ability.

**Conclusions:**

Our results demonstrate a novel mechanism involved in the anti-HIV-1 function of MOV10. Given that both MOV10 and A3G belong to the interferon antiviral system, their synergistic inhibition of HIV-1 suggests that these proteins may play complicated roles in antiviral functions.

## Background

Cellular apolipoprotein-B-mRNA-editing enzyme catalytic polypeptide-like 3G (APOBEC3G or A3G) is a potent antiviral host factor that can be packaged into HIV-1 virions and induces a C–U conversion in the newly synthesized minus-stranded viral DNA, thereby triggering the breakage of viral DNA or generating G-to-A hypermutations that result in a premature stop codon or mutated viral protein [1–5]. HIV-1 virion infectivity factor (Vif) can effectively counteract the antiviral activity of A3G by inducing its degradation through the ubiquitin–proteasome system (UPS) [6–10]. Vif interacts with A3G through its N-terminal domain and has a SOCS-box motif within its C-terminal domain, which includes a BC-box and Cullin-box and interacts with Cullin 5, ElonginB, and ElonginC to form an E3 ubiquitin ligase complex and subsequently mediate the ubiquitination of A3G. CBF-β can bind with Vif directly and facilitate the degradation of A3G. Moreover, CBF-β can increase the stability of HIV-1 Vif and promote assembly of Vif-Cullin 5-E3-ubiquitin-ligase complex; however, ElonginB and ElonginC facilitate the binding of CBF-β with Vif [11–16].

MOV10 is originally identified in the MOV-10 mouse strain, which carries the Moloney murine leukemia virus. It is a member of the UPF1p family and has ATP-dependent 5′–3′ RNA helicase activity [17, 18]. MOV10 has complicated functions and features. For example, MOV10 is found to interact with Argonaute proteins and plays a role in microRNA (miRNA)-mediated regulation [19, 20]. MOV10 is also involved in polycomb-mediated repression of the tumor-suppressor INK4a [21]. Moreover, it has been reported that MOV10 is a type I interferon stimulated gene and several reports have indicated that this protein has broad antiretroviral activity against various viruses, such as HIV-1, murine leukemia virus (MLV), and equine infectious anemia virus (EIAV) [22–25]. Its inhibitory effect on LINE-1 retrotransposition has also been investigated [26, 27]. MOV10 can also be packaged into HIV-1 particles and affects HIV-1 replication at multiple stages [22, 28–30]. MOV10 expresses in varieties of human cells. And according to the data from GEO profile, we found that the expression profile of MOV10 is in moderate or high level in CD4 + T cells and monocytes (https://www.ncbi.nlm.nih.gov/geoprofiles, GEO Profiles ID: 89710126, 106167926, 52933168, 51070326).

Recently, several studies have reported that MOV10 interacts with A3G and both of these proteins are located in P-bodies and can be induced by interferon-α [23, 29, 31]. Based on the similar features of MOV10 and A3G, a research group has studied the possible relationship between these two restriction factors in HIV-1 infection [29]. They co-expressed MOV10 with A3G, but failed to find any functional synergistic effects on viral replication. Conversely, after knocking down endogenous MOV10 by siRNA in the presence of A3G, they did not find any significant impact on HIV-1 infectivity. Nevertheless, they detected the possible synergy of these two inhibitors in the absence of HIV-1 Vif protein. Given that MOV10 can bind with A3G, we hypothesize that MOV10 may affect the process of Vif-mediated degradation of A3G.

Thus, in this study, we aim to elucidate the correlations between MOV10 and A3G in the presence of Vif, which could occur during natural infection by HIV-1. Our findings provide important insights into the role of MOV10 in Vif-mediated A3G degradation and the mechanism through which MOV10 mediates the functional assembly of the Vif-CBF-β-Cullin 5-ElonginB-ElonginC complex to affect the Vif-medicated ubiquitin–proteasome pathway.

## Results

### MOV10 counteracts Vif-mediated degradation of A3G by interfering with the ubiquitin–proteasome pathway

**Fig. 1:**
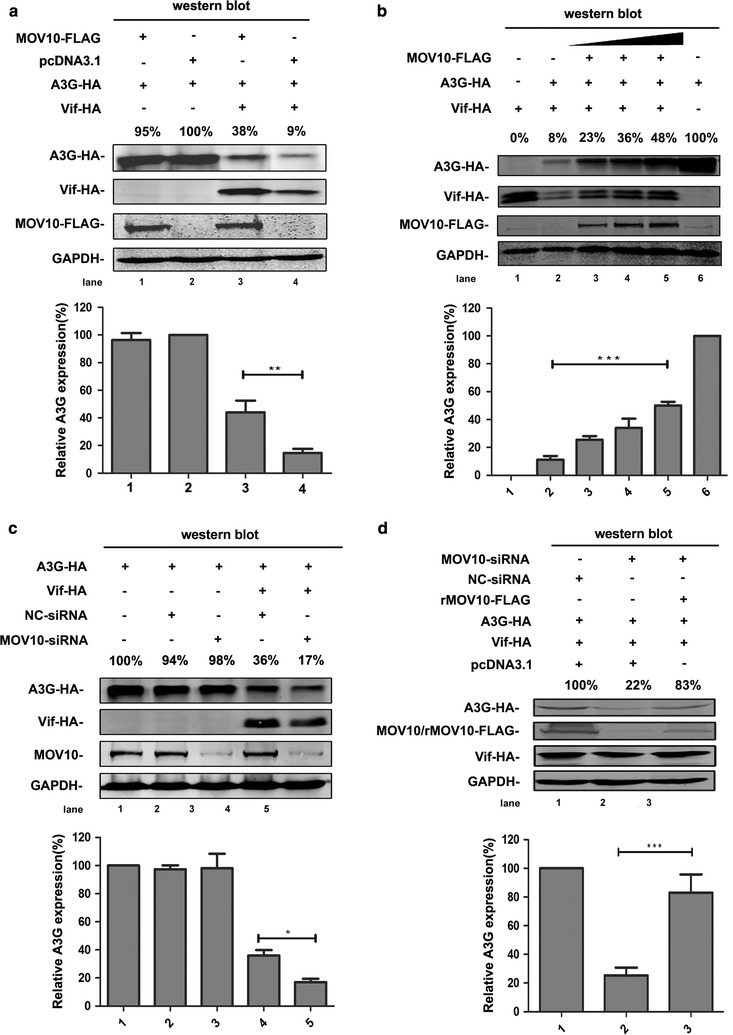
MOV10 counteracts Vif-mediated degradation of A3G. **a**, **b** MOV10 overexpression inhibits Vif-mediated A3G degradation. Human 293T cells were transfected with pcDNA3.1-A3G-HA (0.8 μg), pcDNA3.1-Vif-HA (0.5 μg), and pcDNA3.1-MOV10-FLAG (1.5 μg) (**a**) or different amounts of pcDNA3.1-MOV10-FLAG (from 0.5 to 2 μg) (**b**). Then, cells were collected and lysed at 48 h, and analyzed by western blotting with anti-HA, anti-FLAG, and anti-GAPDH antibodies. **c**, **d** The effect of MOV10 depletion on Vif-mediated degradation of A3G. Cells were transfected with pcDNA3.1-A3G-HA (0.8 μg), pcDNA3.1-Vif-HA (0.5 μg), *MOV10*-specific siRNA (50 nM) (**c**) and/or siRNA-resistant MOV10 construct (rMOV10-FLAG) (0.4 μg) (**d**). After 48 h, cells were collected and analyzed by western blotting assay with anti-HA, anti-FLAG, anti-MOV10 and anti-GAPDH antibodies. Empty vector pcDNA3.1 was used in each transfection to normalize DNA amounts. Values in **a**–**d** represent percentages of A3G or MOV10 normalized against GAPDH and compared with control. The *bar graphs* represent the average expression of A3G with different treatment and relative to the A3G-only reaction control (set to 100%). All the data represent mean ± SD from three independent experiments. Statistical significance was determined using *t* test: *p ≤ 0.05; **p ≤ 0.01; ***p ≤ 0.001

In order to examine the relationships among MOV10, A3G, and Vif, we co-transfected MOV10-FLAG-, A3G-HA-, and Vif-HA-expressing plasmids into 293T cells, and then evaluated the expression levels of A3G and Vif. Interestingly, significant increases in A3G and Vif protein expression were observed in cells overexpressing MOV10 (Fig. 1a). To confirm this phenomenon, we analyzed changes in expression of A3G and Vif in the presence of different levels of MOV10. We found that the enhancement of A3G and Vif expression was correlated with the level of MOV10 (Fig. 1b). We also observed the same phenotype by depleting endogenous MOV10 with *MOV10*-specific siRNA (Fig. 1c) [32]. To exclude the possibility of off-target effects of siRNA, a restoration experiment was conducted. Co-transfection of rMOV10-FLAG-expressing plasmid, a MOV10 construct that is resistant to siRNA-targeting, with the *MOV10*-specific siRNA restored the expression of A3G (Fig. 1d), indicating that *MOV10*-specific siRNA does not have off-target effects.

**Fig. 2:**
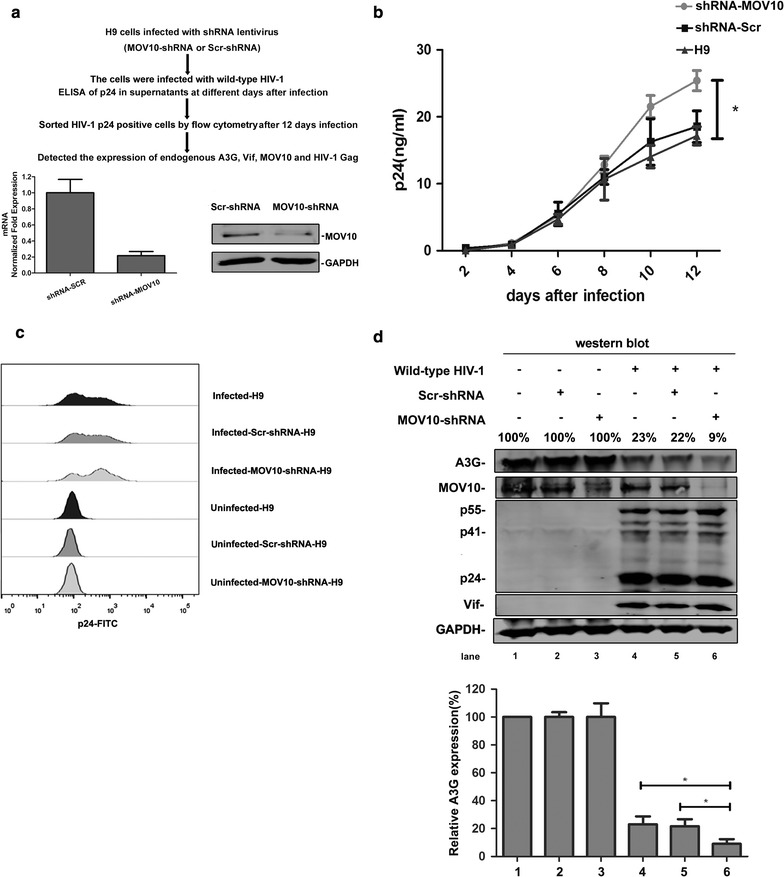
MOV10 protects A3G from Vif-mediated degradation in wild-type HIV-1. **a** H9 cells were infected with pLKO.1-MOV10-shRNA or pLKO.1-Scr-shRNA lentivirus for 8 h and then selected with puromycin for 2 weeks. MOV10-knockdown H9 cells and control cells were infected with wild-type HIV-1 for 3 h and cultured with fresh medium for 12 days. The culture supernatants were collected at the indicated time points. Then HIV-1 p24 was detected using HIV-1 p24 ELISA kit at different time points (**b**). And at 12th day, these cells were analyzed by flow cytometer (**c**). HIV-1 p24 positive H9 cells were sorted and detected by western blotting with anti-MOV10, anti-A3G, anti-Vif, anti-HIV-1 p24, and anti-GAPDH antibodies (**d**). Values in **d** represent percentages of A3G or MOV10 normalized against GAPDH and compared with control. The *bar graphs in* ***d*** represent the average expression of A3G with different treatment and relative to the A3G-only reaction control (set to 100%). Data in **a**, **b**, and **d** represent mean ± SD from three independent experiments. *, statistically significant, *p* ≤ 0.05 (*t* test). All the results are representative of at least three independent experiments

To further validate this phenotype, a similar experiment was performed in H9 cells infected with wild-type HIV-1. MOV10-knockdown H9 cells were constructed by the infection of MOV10-specific shRNA-expressing lentivirus. The cells were then infected with wild-type HIV-1 viruses. The culture supernatants were collected at different days after infection, and HIV-1 p24 was detected by ELISA kit. After culture for 12 days, HIV-1 p24-positive cells were sorted by flow cytometry (Fig. 2). In these HIV-1 p24-positive cells, the expression of endogenous A3G was significantly down-regulated by endogenous MOV10 depletion. HIV-1 replication and the expression of Gag protein were also enhanced by the depletion of MOV10 (Fig. 2d). The results demonstrate that MOV10 and A3G synergistically inhibit the replication of HIV-1.

**Fig. 3:**
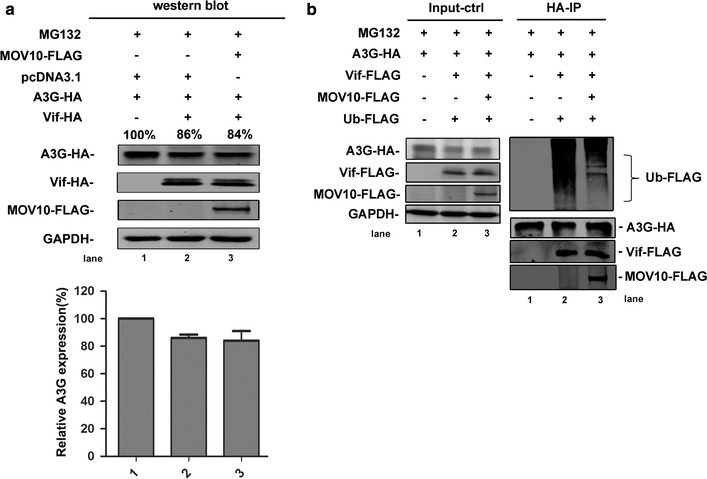
MOV10 prevents A3G from Vif-induced degradation by decreasing the ubiquitination of A3G. **a** Human 293T cells were transfected with pcDNA3.1-A3G-HA (0.8 μg), pcDNA3.1-Vif-HA (0.5 μg), and pcDNA3.1-MOV10-FLAG (1.5 μg) and then treated with MG132 (4 μM) for 16 h. Lysed cells were collected at 48 h and detected by western blotting with anti-HA, anti-FLAG, and anti-GAPDH antibodies. **b** 293T cells were transfected with pcDNA3.1-A3G-HA (2 μg), pcDNA3.1-Vif-FLAG (1.25 μg), pcDNA3.1-MOV10-FLAG (2.5 μg), and pcDNA3.1-Ub-FLAG (3 μg). Cells were treated with MG132 (4 μM) for 16 h and analyzed by co-immunoprecipitation with anti-HA agarose beads. And then, samples were detected by western-blotting using anti-HA, anti-FLAG, and anti-GAPDH. Values in **a** represent percentages of A3G normalized against GAPDH and compared with control. The *bar graphs in* **a** represent the average expression of A3G with different treatment and relative to the A3G-only reaction control (set to 100%). Data in **a** represent mean ± SD from three independent experiments. Empty vector pcDNA3.1 was used to equalize DNA amounts in each transfection. Data in **a** and **b** are representative of at least three independent experiments

Considering the relationships between Vif/A3G and the ubiquitin–proteasome pathway [6–8, 10, 33], we evaluated the effects of MOV10 on the expression levels of A3G and Vif in the presence of the proteasome inhibitor MG132. After treatment with MG132 for 16 h, the expression levels of A3G and Vif were not affected by MOV10 overexpression (Fig. 3a). Further study showed that MOV10 could decrease the ubiquitination of A3G directly (Fig. 3b). Taken together, these data indicate that MOV10 can protect A3G from Vif-mediated degradation by interfering with the ubiquitin–proteasome pathway.

### MOV10 affects the assembly of the Vif-CBF-β-Cullin 5-ElonginB-ElonginC complex

**Fig. 4:**
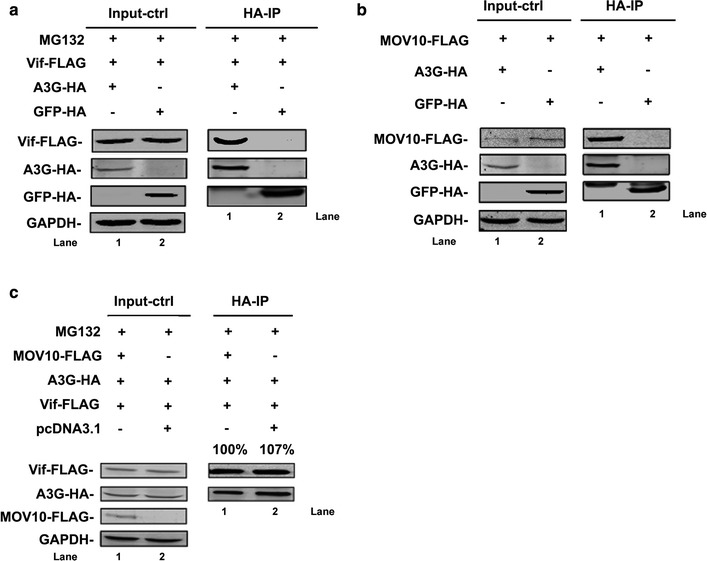
MOV10 has no influence on the binding of A3G with Vif. **a**, **b** A3G interacts with Vif or MOV10 effectively. **a** Human 293T cells were transfected with 2 μg of pcDNA3.1-A3G-HA (pcDNA3.1-GFP-HA as a control) and 1 μg of pcDNA3.1-Vif-FLAG and then treated with MG132 for 16 h. **b** Human 293T cells were transfected with 1 μg of pcDNA3.1-Vif-FLAG and 2 μg of pcDNA3.1-A3G-HA or pcDNA3.1-GFP-HA. **a**, **b** lysates from these transfected cell samples were subjected to co-immunoprecipitation analysis using anti-HA agarose beads and then detected by western blotting. **c** The effect of MOV10 on the interaction between A3G and Vif. 293T cells were transfected with 2 μg of pcDNA3.1-A3G-HA together with 1 μg of pcDNA3.1-Vif-FLAG, and 2 μg of pcDNA3.1-MOV10-FLAG and then treated with MG132 (4 μM) for 16 h. Samples were immunoprecipitated with anti-HA agarose beads and analyzed by western blotting. Empty vector pcDNA3.1 was used to equalize DNA amounts in each transfection. Values in **c** represent portions of Vif-FLAG normalized against A3G-HA relative to control values. Data in **a**, **b**, and **c** are representative of at least three independent experiments

Previous studies have reported that A3G can bind with Vif (Fig. 4a) [10]. And, A3G protein contains two domains: the N-terminal domain is responsible for encapsidation and the C-terminal domain is responsible for deamination activity [34, 35]. Only the N-terminal domain of A3G can bind with Vif and the binding initiates the degradation process of A3G [36]. Because A3G can also interact with MOV10 (Fig. 4b) [20], the interaction of Vif with A3G may be affected by MOV10. To this end, we investigated the effects of MOV10 on the interaction between Vif and A3G in the presence of MG132. Human 293T cells were transfected with MOV10-FLAG-, A3G-HA-, and Vif-FLAG-expressing plasmids and then treated with MG132 for 16 h. However, we did not detect any changes in the levels of Vif-FLAG in the A3G-HA-immunoprecipitated samples with or without MOV10 (Fig. 4c). These results suggest that MOV10 does not affect the binding of Vif with A3G and therefore it may interfere with other steps in the A3G degradation process.

**Fig. 5:**
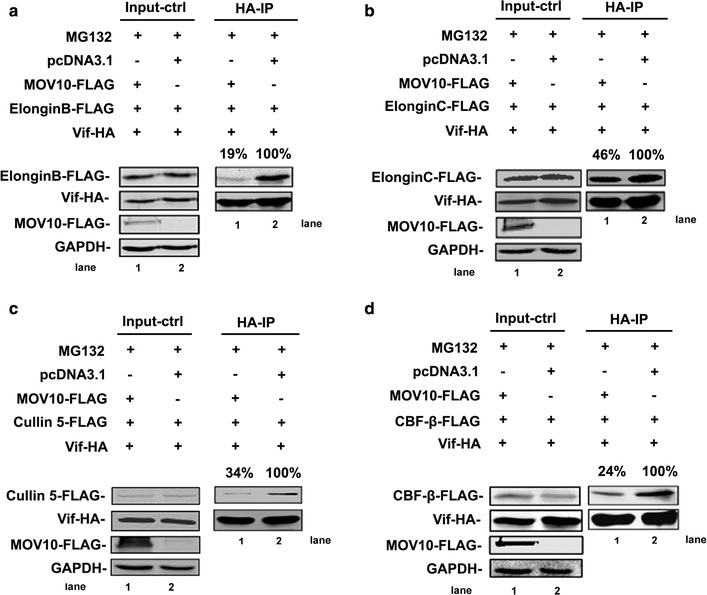
MOV10 affects the assembly of Vif-CBF-β-Cullin 5-ElonginB-ElonginC Complex. **a**–**d** The effect of MOV10 on the interaction between Vif and ElonginB (**a**), ElonginC (**b**), Cullin 5 (**c**), or CBF-β (**d**). 293T cells were transfected with pcDNA3.1-MOV10-FLAG (2 μg), pcDNA3.1-Vif-HA (1 μg), and 4 μg of ElonginB-FLAG (**a**) or ElonginC-FLAG (**b**) or pcDNA3.1-Cullin 5-FLAG (**c**) or pcDNA3.1-CBF-β-FLAG (**d**). After treated with MG132 (4 μM) for 16 h, cell lysates were immunoprecipitated with anti-HA agarose beads and analyzed by immunoblotting using anti-FLAG, anti-HA, and anti-GAPDH antibodies. In each transfection, empty vector pcDNA3.1 was used to normalize DNA amounts. Values in **a**–**d** represent percentages of ElonginB-FLAG/ElonginC-FLAG/Cullin 5-FLAG/CBF-β-FLAG normalized against Vif-HA relative to control values. All the data is representative of at least three independent experiments

The interaction of Vif with CBF-β, Cullin 5, ElonginB, and ElonginC can facilitate the formation of a ubiquitin ligase complex, which is required for Vif to induce the degradation of A3G [11–13, 37, 38]. Therefore, we next examined the effects of MOV10 on the interaction between Vif and different components in the complex. We transfected Vif-HA- and MOV10-FLAG-expressing plasmids with pcDNA3.1-ElonginB-FLAG, pcDNA3.1-ElonginC-FLAG, pcDNA3.1-Cullin 5-FLAG, or pcDNA3.1-CBF-β-FLAG into 293T cells. After immunoprecipitation, we found that the interactions of Vif with ElonginB, ElonginC, Cullin 5, and CBF-β significantly decreased when MOV10 was overexpressed (Fig. 5a–d), indicating that MOV10 affects Vif-ElonginB, Vif-ElonginC, Vif-Cullin 5, and Vif-CBF-β interactions during the assembly of the Vif-CBF-β-Cullin 5-ElonginB-ElonginC complex.

**Fig. 6:**
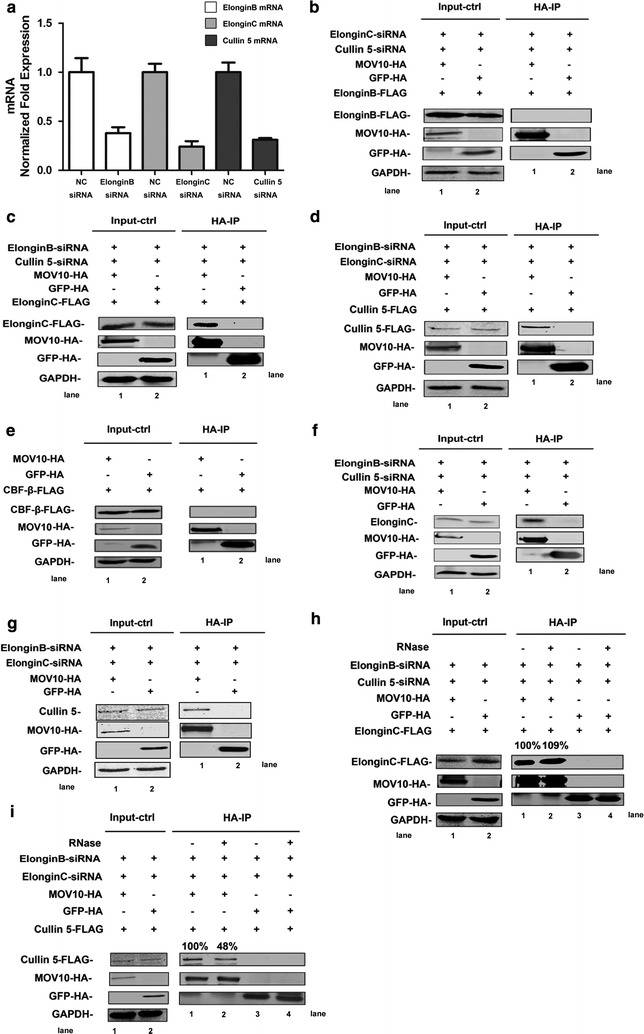
MOV10 binds with ElonginC or Cullin 5. **a** The knockdown efficiency of siElonginB, siElonginC and siCullin 5. 293T cells were transfected with siElonginB, siElonginC or siCullin 5, after 48 h, the cells were collected and detected with qRT-PCR. Data in A represents mean ± SD (*error bars*). **b**–**i** Co-immunoprecipitated analysis of the interaction between MOV10 and ElonginB (**b**), ElonginC (**c**, **f**, **h**), Cullin 5 (**d**, **g**, **i**), or CBF-β (**e**). pcDNA3.1-ElonginB-FLAG plus siElonginC and siCullin 5 (**b**), pcDNA3.1-ElonginC-FLAG plus siElonginB and siCullin 5 (**c**, **h**), pcDNA3.1-Cullin 5-FLAG plus siElonginB and siEloingC (**d**, **i**) or pcDNA3.1-CBF-β-FLAG (**e**) was transfected into 293T cells with pcDNA3.1-MOV10-HA or pcDNA3.1-GFP-HA. 293T cells were transfected with pcDNA3.1-MOV10-HA (pcDNA3.1-GFP-HA as a control) plus siElonginB and siCullin 5 (**f)** or siElonginB and siEloingC (**g**). After 48 h, the cells were collected and immunoprecipitated with anti-HA agarose beads (**b**–**i**). The samples in **h** and **i** were treated with RNase mixture. And then, immunoprecipitated samples were analyzed by immunoblotting with anti-FLAG, anti-HA, anti-GAPDH, anti-MOV10, anti-ElonginC, and anti-Cullin 5 antibodies. Empty vector pcDNA3.1 was used to equalize DNA amounts in each transfection. Values in **h** and **i** represent portions of ElonginC-FLAG/Cullin 5-FLAG normalized against MOV10-HA and compared with control. Data in **a**–**i** is representative of at least three independent experiments

According to the above results, we suspected that MOV10 could interact with ElonginB, ElonginC, Cullin 5, or CBF-β. To test this hypothesis, we co-transfected 293T cells with pcDNA3.1-MOV10-HA plus pcDNA3.1-ElonginB-FLAG, pcDNA3.1-ElonginC-FLAG, pcDNA3.1-Cullin 5-FLAG or pcDNA3.1-CBF-β-FLAG. Previous study has demonstrated that ElonginC, ElonginB, and Cullin 5 can interact with each other [39]. To eliminate the influence of these endogenous proteins, siRNAs specific to *ElonginB*, *ElonginC*, and *Cullin 5* mRNA were also co-transfected into cells at the same time (Fig. 6a). After immunoprecipitation and western blotting, significant binding was found between MOV10 and ElonginC or Cullin 5 (Fig. 6c, d). To further confirm the binding, we detected the interaction between MOV10-HA and endogenous ElonginC or Cullin 5. As shown in the Fig. 6f, g, the same phenomenon was observed. After treatment with an RNase mixture, we found that the binding of MOV10 with Cullin 5 was partially dependent on RNA, whereas the interaction between MOV10 and ElonginC was not (Fig. 6h, i). However, the interaction between MOV10 and ElonginB or CBF-β was not detected (Fig. 6b, e).

### The helicase activity center of MOV10 is required for its inhibitory effects on Vif-mediated A3G degradation

**Fig. 7:**
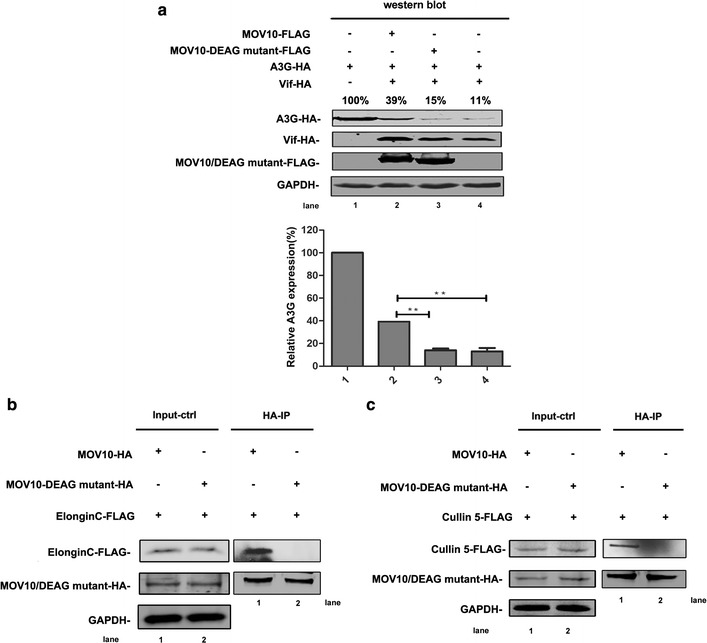
The DEAG-box motif of MOV10 is required for the binding of MOV10 with ElonginC or Cullin 5. **a** The effect of MOV10-DEAG mutant on Vif-mediated A3G degradation. 293T cells were transfected with 0.4 μg of pcDNA3.1-Vif-HA, 0.8 μg of pcDNA3.1-A3G-HA, and 1.5 μg of pcDNA3.1-MOV10-FLAG or pcDNA3.1-MOV10-DEAG-mutant-FLAG as indicated. After 48 h, cell lysates were detected by western blotting assay with anti-HA, anti-FLAG, and anti-GAPDH antibodies. Values represent portions of A3G-HA normalized against GAPDH and compared with control. **b**, **c** Co-immunoprecipitated analysis of the interaction between MOV10-DEAG mutant and ElonginC or Cullin 5. Human 293T cells were transfected with 2 μg of pcDNA3.1-MOV10-HA or pcDNA3.1-MOV10-DEAG-HA and 6 μg of pcDNA3.1-ElonginC-FLAG or pcDNA3.1-Cullin 5-FLAG. After 24 h, MG132 were added in the transfected cells for 16 h. Then, the cells were collected for co-immunoprecipitation analysis with anti-HA agarose beads and detected by western blotting with anti-HA, anti-FLAG, and anti-GAPDH antibodies. In each transfection, empty vector pcDNA3.1 was used to equalize DNA amounts. Values in **a** represent percentages of A3G-HA normalized against GAPDH relative to control. The *bar graphs* in **a** represent the average expression of A3G with different treatments and relative to the A3G-only reaction control (set to 100%). Data in **a** represent mean ± SD from three independent experiments. Statistical significance was determined using *t* test: **p ≤ 0.01. All the data in **a**, **b**, and **c** is representative of at least three independent experiments

MOV10 contains a DEAG-box (D-E-A-G = Asp-Glu-Ala-Gly) motif and the DEAG-box mutant impairs the helicase activity of MOV10 [18, 28]. To examine whether the DEAG-box motif was required for the effects of MOV10 on Vif-mediated degradation of A3G, we used a MOV10-DEAG mutant (a point mutation in the DEAG-box motif, from DEAG to DQAG) to repeat the experiment shown as Fig. 1a [18, 22, 23, 32, 40]. Compared with wild-type MOV10, the MOV10-DEAG mutant almost lost the ability to prevent the degradation of A3G mediated by Vif (Fig. 7a), suggesting that the DEAG-box motif is involved in regulating this inhibitory effects of MOV10. To confirm this conclusion, we further detected the binding of the MOV10-DEAG mutant with ElonginC or Cullin 5. Compared with wild-type MOV10, the bindings of MOV10-DEAG mutant with ElonginC or Cullin 5 decreased significantly (Fig. 7b, c), suggesting that the DEAG-box motif of MOV10 plays an important role in the interaction between MOV10 with ElonginC or Cullin 5.

### MOV10 counteracts Vif-mediated A3G degradation in the context of HIV-1 replication

**Fig. 8:**
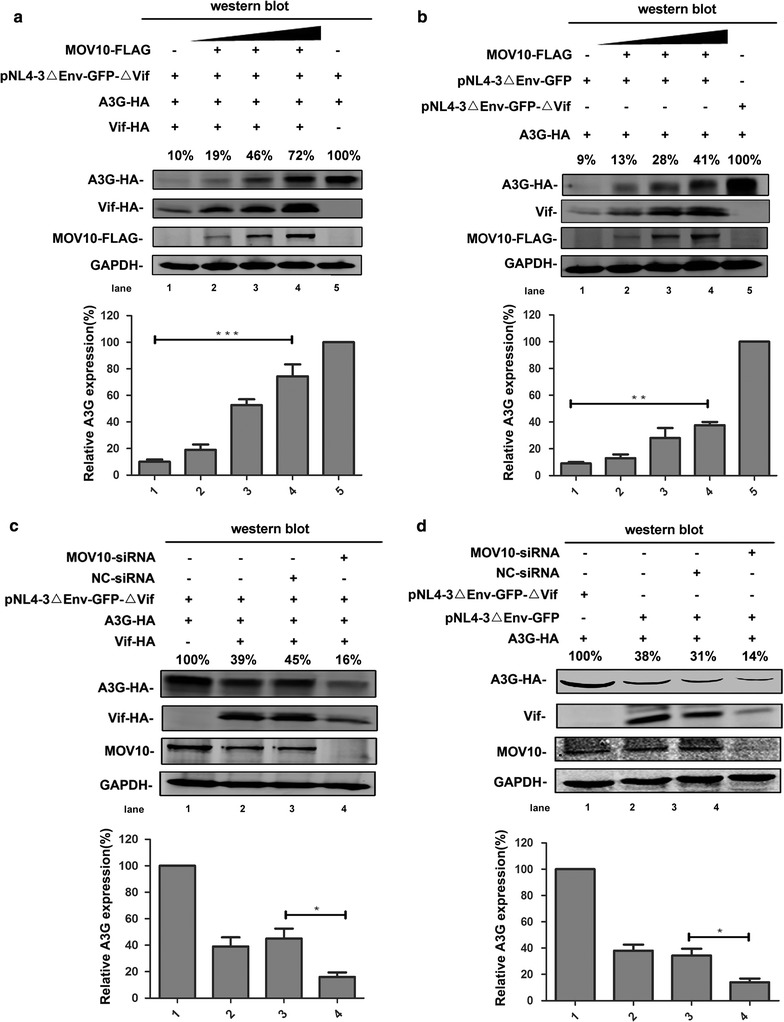
MOV10 reduces A3G proteasomal degradation significantly in the context of HIV-1 replication. **a**, **b** Overexpression of MOV10 inhibits Vif-induced A3G degradation in the context of HIV-1 replication. Human 293T cells were transfected with 0.8 μg of pcDNA3.1-A3G-HA, different amounts of pcDNA3.1-MOV10-FLAG (from 0.5 μg to 2 μg), 0.5 μg of pcDNA3.1-Vif-HA, 1 μg of pNL4-3ΔEnv-GFP-ΔVif (**a**) and/or 1 μg of pNL4-3ΔEnv-GFP (**b**) as indicated. Then, cells were collected at 48 h for western blotting assay with anti-FLAG, anti-HA, anti-Vif, and anti-GAPDH antibodies. **c**, **d** The effect of MOV10 depletion on the proteasomal degradation of A3G in the context of other HIV-1 proteins. 293T cells were transfected with pcDNA3.1-A3G-HA (0.8 μg), pcDNA3.1-Vif-HA (0.5 μg), *MOV10*-specific siRNA (or negative control-siRNA), 1 μg of pNL4-3ΔEnv-GFP-ΔVif (**c**) and/or 1 μg of pNL4-3ΔEnv-GFP (**d**). Cell lysates were detected by immunoblotting with anti-HA, anti-FLAG, anti-MOV10, anti-Vif, and anti-GAPDH antibodies. The *bar graphs* represent the average expression of A3G with different treatment and relative to the A3G-only reaction control (set to 100%). Data in **a**–**d** represent mean ± SD from three independent experiments. Statistical significance was determined using *t* test: *p ≤ 0.05; **p ≤ 0.01; ***p ≤ 0.001. Empty vector pcDNA3.1 was used to equalize DNA amounts in each transfection. Values in **a**–**d** represent portions of A3G-HA normalized against GAPDH and compared with control. Each data is representative of at least three independent experiments

All of the above experiments were performed in the context of lack of other HIV-1 proteins. To verify whether the effect of MOV10 on Vif-mediated A3G degradation could be observed in the context of HIV-1 replication, we used two types of HIV-1 pNL4-3ΔEnv-GFP clones. Human 293T cells were transfected with pNL4-3ΔEnv-GFP-ΔVif, pcDNA3.1-Vif-HA, pcDNA3.1-A3G-HA, and different amounts of MOV10-FLAG-expressing plasmid. Consistent with Fig. 1b, the expression levels of A3G and Vif were correlated with the expression levels of MOV10 in the presence of other HIV-1 proteins (Fig. 8a). Moreover, the same results were observed when we co-transfected 293T cells with pNL4-3ΔEnv-GFP, pcDNA3.1-A3G-HA, and different amounts of pcDNA3.1-MOV10-FLAG (Fig. 8b). To further confirm this, we examined the effect of MOV10 depletion on Vif-mediated A3G degradation in the context of HIV-1 replication. The same phenotypes as shown in Fig. 1c were recapitulated by *MOV10*-specific siRNAs (Fig. 8c, d). These data indicate that MOV10 can inhibit Vif-mediated degradation of A3G in the context of HIV-1 replication.

### MOV10 increases the quantity of A3G in HIV-1 virions by protecting A3G from Vif-mediated degradation

**Fig. 9:**
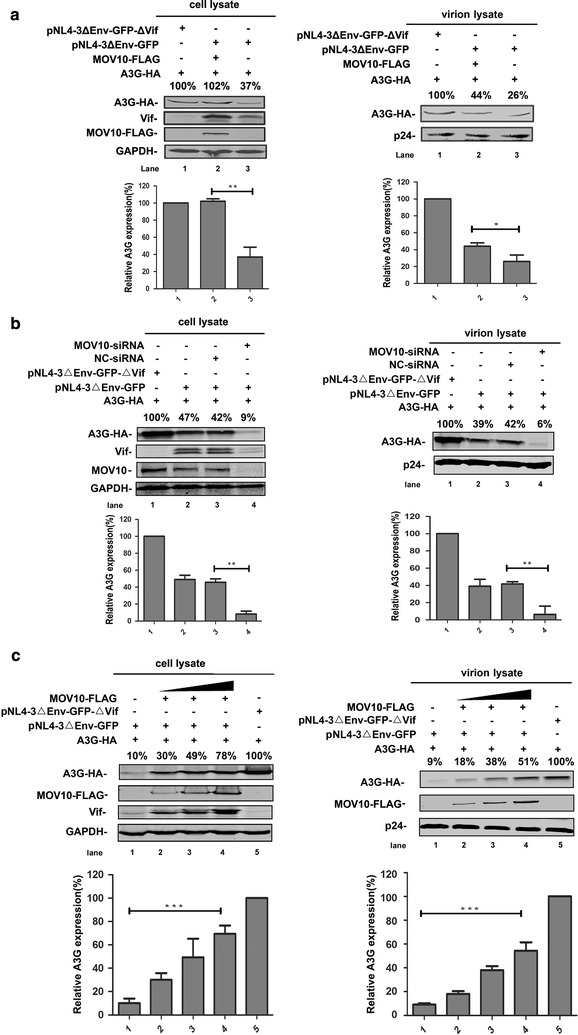
MOV10 increases the quantity of A3G in HIV-1 virions. **a** Overexpression of MOV10 increases the quantity of A3G in virions. Human 293T cells were transfected with 0.8 μg of pcDNA3.1-A3G-HA, 1.5 μg of pcDNA3.1-MOV10-FLAG, 1 μg of pNL4-3ΔEnv-GFP or pNL4-3ΔEnv-GFP-ΔVif as indicated. **b** The effect of endogenous MOV10 knockdown on Vif-induced A3G degradation in HIV-1 virions. Cells were transfected with 0.8 μg of pcDNA3.1-A3G-HA, 50 nM of *MOV10*-specific siRNA (or negative control siRNA), and 1 μg of pNL4-3ΔEnv-GFP or pNL4-3ΔEnv-GFP-ΔVif. **c** MOV10 can be packaged into HIV-1 virions and its packaging level increases with the survival level of A3G. 293T cells were transfected with 0.8 μg of pcDNA3.1-A3G-HA, different amounts of pcDNA3.1-MOV10-FLAG (from 0.5 to 1.5 μg), 1 μg of pNL4-3ΔEnv-GFP or pNL4-3ΔEnv-GFP-ΔVif. **a**, **b** and **c** After 48 h, cell pellets and supernatants were collected respectively. Cell pellets were lysed and subjected to immunoblotting with anti-HA, anti-FLAG, anti-MOV10, anti-Vif, and anti-GAPDH antibodies. VLPs were collected from filtered supernatants by ultracentrifugation. The pelleted VLPs were lysed and detected by western blotting with anti-HA, anti-FLAG, and anti-p24 antibodies. The *bar graphs* represent the average expression of A3G with different treatments and relative to the A3G-only reaction control (set to 100%). Data in **a**–**c** represent mean ± SD from three independent experiments. Statistical significance was determined using *t* test: *p ≤ 0.05; **p ≤ 0.01; ***p ≤ 0.001. In each transfection, empty vector pcDNA3.1 was used to equalize DNA amounts. Values in **a**–**c** represent percentages of A3G-HA normalized against GAPDH or p24 and compared with control. Results are representative of at least three independent experiments

As noted above, we demonstrated that MOV10 could increase the quantity of A3G by interfering with the proteasome pathway in virus-producing cells. Given that A3G can be packaged into HIV-1 virions and exert anti-HIV-1 activity [2, 35, 41–44]. We next evaluated the effects of MOV10 on the quantity of A3G in HIV-1 virions. We co-transfected 293T cells with pNL4-3ΔEnv-GFP, pcDNA3.1-A3G-HA, and pcDNA3.1-MOV10-FLAG, subsequently collected the supernatants and cells of each sample. Interestingly, although MOV10 enhanced A3G levels in cell lysates, the quantity of A3G was also increased in the supernatant viral particles (Fig. 9a). To further confirm this, the A3G levels were analyzed in virus-producing cells and viral particles following depletion of endogenous MOV10 with *MOV10*-specific siRNAs. MOV10 knockdown could reduce the quantity of A3G in both virus producing cells and viral particles (Fig. 9b). Previous studies showed that MOV10 can be packaged into virions and affects HIV-1 replication at multiple stages. Here, we overexpressed MOV10 in a dose-dependent manner, and we found that there was a synergy between the packaging levels of MOV10 and A3G (Fig. 9c). Taken together, these results demonstrate that MOV10 increases the quantity of A3G in HIV-1 virions by interfering with the Vif-mediated ubiquitin–proteasome pathway.

**Fig. 10:**
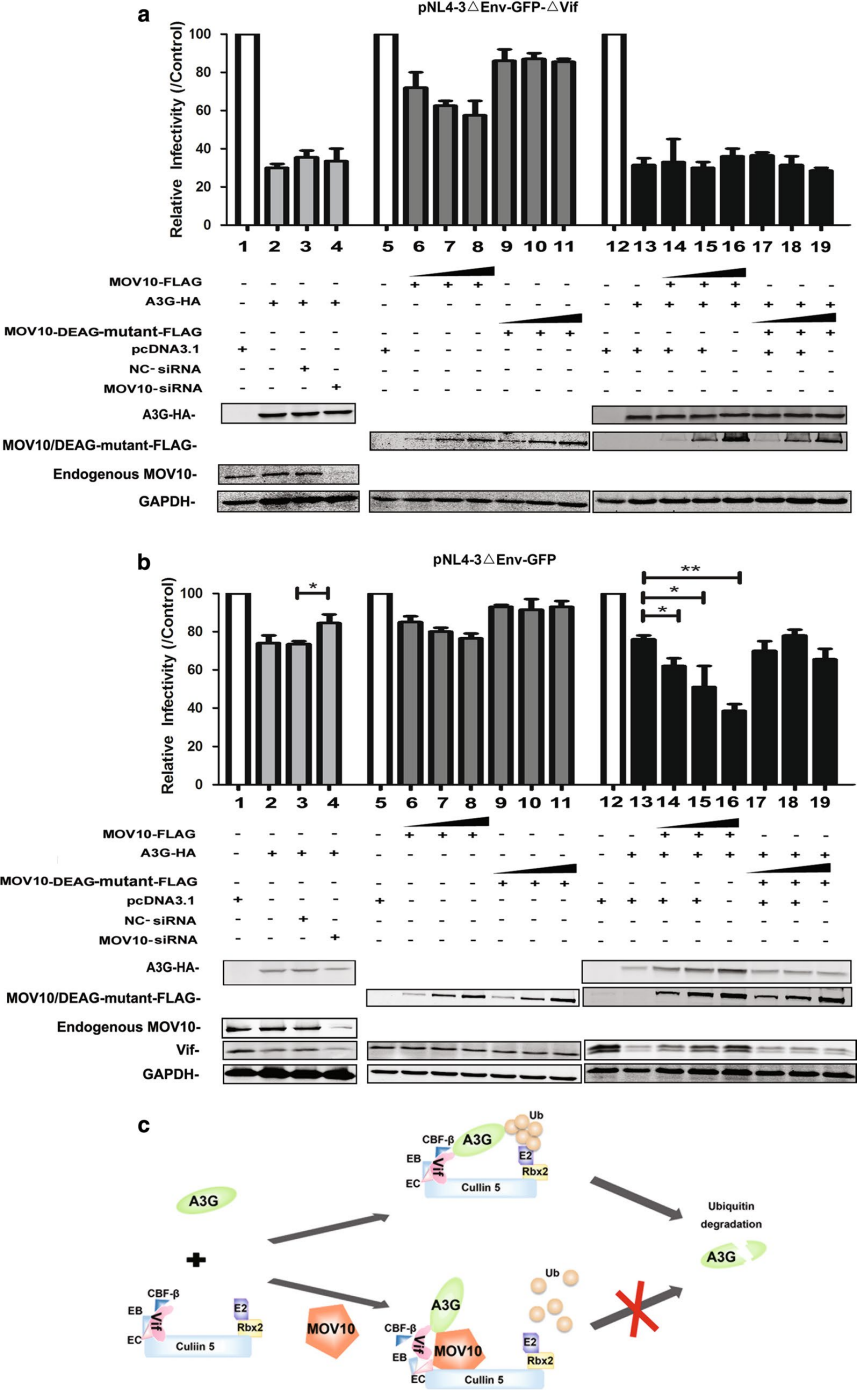
MOV10 synergistically enhances the inhibitory effect of A3G on the infectivity of HIV-1. **a**, **b** *MOV10*-specific siRNA was transfected in 293T cells with pcDNA3.1-A3G-HA (0.8 μg), pCMV-VSV-G (2.5 μg), and pNL4-3ΔEnv-GFP-ΔVif (7.5 μg) or pNL4-3ΔEnv-GFP (7.5 μg). 293T cells were co-transfected with pCMV-VSV-G (2.5 μg), pNL4-3ΔEnv-GFP-ΔVif (7.5 μg) or pNL4-3ΔEnv-GFP (7.5 μg), and increasing amounts of pcDNA3.1-MOV10-FLAG (0.5–1.5 μg) or pcDNA3.1-MOV10-DEAG-mutant-FLAG (0.5–1.5 μg) in the presence or absence of pcDNA3.1-A3G-HA (0.8 μg). Culture supernatants containing 5 ng of p24 were used to infect TZM-bl cells and luciferase activity was determined at 72 h post infection. For viruses with different amounts of MOV10 but no A3G, and viruses with a fixed amount of A3G combined with different amounts of MOV10 or not, the data are plotted as relative infectivity, with the control virus (pcDNA3.1) set to 100%. Error bars represent standard errors from three independent experiments. Statistical significance was determined using *t* test: *p ≤ 0.05; **p ≤ 0.01. **c** A cartoon to show the interaction between MOV10, A3G, and Vif. In the absence of MOV10, the Vif-CBF-β-Cullin 5-ElonginB-ElonginC complex is stable and triggers the proteasomal degradation of A3G. In the presence of MOV10, the assembly of Vif-CBF-β-Cullin 5-ElonginB-ElonginC complex can be disturbed by MOV10, which leads to A3G escaping from Vif-induced proteasomal degradation

Furthermore, to explore the potential synergistic effect of MOV10 and A3G on the infectivity of the newly-produced virions, NL4-3-ΔEnv-GFP and NL4-3-ΔEnv-GFP-ΔVif particles were produced with increasing amounts of MOV10 in either the presence or absence of A3G. After normalization for HIV-1 p24, TZM-bl cells were infected with these viral particles and then the infectivity of viruses was determined (Fig. 10a, b). For NL4-3-ΔEnv-GFP-ΔVif particles, compared with the group of single MOV10 or A3G treatment, the inhibitory effect of MOV10 or MOV10-DEAG mutant plus A3G group was equal to the effect of single A3G treatment group. And, the depletion of endogenous MOV10 with siRNA in the presence of A3G also has no impact on HIV-1 infectivity. It is consistent with previous study that co-expression of MOV10 did not enhance the inhibitory effect of A3G on the infectivity of ΔVif HIV-1 (Fig. 10a) [29]. However, for NL4-3-ΔEnv-GFP particles, the fold reductions in infectivity at various amounts of MOV10 were different in the presence or absence of A3G and endogenous MOV10 was helpful for A3G to decrease the infectivity of HIV-1. But, MOV10-DEAG mutant lost its ability to help A3G decrease the infectivity of HIV-1 (Fig. 10b). This data further confirms that DEAG-box motif of MOV10 plays an important role in protecting A3G from Vif-mediated degradation. These results indicate that the inhibitory effect of A3G on the infectivity of HIV-1 can be synergistically enhanced by MOV10.

## Discussion

In this report, we studied the relationship between MOV10 and A3G in the presence of HIV-1 Vif. Interestingly, we observed that MOV10 increased the levels of A3G and Vif in a concentration-dependent manner. The phenomenon is consistent with the previous study that the expression levels of A3G and Vif can be increased simultaneously in the presence of the proteasome inhibitor MG132 [45, 46]. Vif is an E3 ubiquitin ligase substrate receptor that interacts with host factors ElonginB, ElonginC, Cullin 5, and CBF-β to form an E3 ubiquitin ligase complex, which results in the polyubiquitylation of both Vif and A3G. And then, the Vif-A3G complex can be degraded together via the ubiquitin–proteasome system [6, 12, 28, 37, 47, 48]. HIV-1 Vif has at least 4 conserved motifs, which are required for interactions with host proteins. The HCCH motif can bind to Cullin 5, the BC-box motif (144-SLQYLA-149) binds to ElonginC, 101-DVMK-104 binds to ElonginB, and 88-EW-89 is crucial for binding with CBF-β [38, 49–51]. Through a series of co-immunoprecipitation analyses, we found that MOV10 can disrupt the interaction of Vif with ElonginB, ElonginC, Cullin 5, or CBF-β and then decrease the ubiquitination of A3G. Finally, the degradations of A3G and Vif are blocked and their expression levels in cells are increased subsequently. These results suggest that MOV10 functions to mediate assembly of the Vif-CBF-β-ElonginB-ElonginC-Cullin 5 complex.

Previous studies have shown that Cullin 5 functions as a scaffold protein for the *E3 ubiquitin ligase* [52, 53] ubiquitin–proteasome . ElonginB, ElonginC, and CBF-β are adaptor proteins that function to maintain this complex. Moreover, Vif acts as a substrate acceptor to modulate the degradation of A3G [52, 54]. Therefore, reduced binding of Vif with Cullin 5 could affect the complex assembly efficiency. Moreover, researchers have verified the interactions between the different components of the complex. The binding of Cullin 5 to Vif enhances the stability of the Vif-CBF-β interaction [55]. Conversely, CBF-β is also crucial for the binding of Vif with Cullin 5, ElonginB, and ElonginC [37, 56, 57]. ElonginB and ElonginC play important roles in the interaction between Vif and CBF-β [16]. To clarify the mechanisms through which MOV10 disrupts the assembly of the Vif-CBF-β-ElonginB-ElonginC-Cullin 5 complex, we examined whether there were direct interactions between MOV10 and different components of the CBF-β-Cullin 5-ElonginB-ElonginC complex. The results demonstrate that MOV10 can bind with ElonginC or Cullin 5 and that binding between MOV10 and Cullin 5 is partially dependent on RNA. Our own study and previous studies have shown that MOV10 usually interacts with numerous RNA-associated proteins, such as AGO1/2, A3G, and HIV-1 Rev [20, 32]. Thus, it is not surprising that MOV10 interacts with Cullin 5 in an RNA-dependent manner. Accordingly, significant decreases in the binding of Vif with ElonginB, ElonginC, Cullin 5, and CBF-β were observed when MOV10 was overexpressed. For the inhibitory effects of MOV10 on the binding of Vif with ElonginB or CBF-β, the interactions of MOV10 with ElonginC, Cullin 5, and Vif may induce structural changes in the Vif-CBF-β-ElonginB-ElonginC-Cullin 5 complex, subsequently disrupting the interactions between Vif and ElonginB and between Vif and CBF-β. Several studies have shown that DEAG-box motif of MOV10 is crucial for its helicase activity [18, 28]. In our report, we also explored the correlation between the helicase activity and anti-HIV-1 function of MOV10. Because the binding of MOV10-DEAG mutant with ElonginC or Cullin 5 decreased significantly, it almost lost the ability to protect A3G from Vif-mediated degradation, indicating that the helicase activity center of MOV10 is required for its inhibitory effect on Vif-mediated A3G degradation.

According to these data, we propose a model that, during the process of HIV-1 infection, MOV10 can interact with ElonginC and Cullin 5 to disturb the interaction of Vif with ElonginB, ElonginC, Culiin 5 or CBF-β and subsequently interfere with the assembly of Vif-CBF-β-Cullin 5-ElonginB-ElonginC complex which induces the ubiquitination of A3G. In this way, MOV10 prevents A3G from proteasomal degradation and subsequently enhances the level of A3G in virus-producing cells. It is well known that A3G can be packaged into HIV-1 virions and inhibit HIV-1 replication at multiple stages, the A3G level in newly-produced virions should be increased in the presence of MOV10 (Fig. 10c) [44]. Indeed, we found that the A3G level was significantly enhanced in virions by MOV10 overexpression and significantly reduced by MOV10 knockdown in the context of HIV-1 replication.

Moreover, the synergistic effects on the infectivity and replication of HIV-1 between MOV10 and A3G have been tested. Previous study has demonstrated that co-expression of MOV10 does not affect the inhibitory effect of A3G on the infectivity of ΔVif HIV-1 [29]. Our results also show the same phenomenon that the inhibitory effect of A3G plus MOV10 group on ΔVif HIV-1 is consistent with the effect of single A3G treatment group. As the anti-HIV-1 activity of A3G is more potent than that of MOV10, it will overspread the anti-HIV-1 effect of MOV10 when Vif deficiency. Nevertheless, consistent with our hypothesis, the infectivity of Vif-positive viral particles is synergistically inhibited by MOV10 and A3G. In 2010, Wang et al. [22] have shown that the replication of HIV-1 was enhanced by the depletion of endogenous MOV10 in permissive human T cell line (CEM-SS). Previous study also showed that MOV10 can be packaged into HIV-1 virions and inhibit viral replication at a postentry step [28]. However, little effect of MOV10 on HIV-1 replication in Hut78 T cells was reported by another group [58]. Considering that high concentration of the virus and short term infection will cover up the true effect of antiviral factors, we used low dose virus (5 ng of HIV-1 p24) to perform the experiment and extended the observation time. We found that the replication of wild-type HIV-1 was enhanced in MOV10-shRNA transduced non-permissive human T cells (H9), indicating that MOV10 and A3G can synergistically inhibit HIV-1 replication.

## Conclusions

Therefore, our results reveal a novel anti-HIV-1 mechanism of MOV10: it prevents A3G from Vif-induced proteasomal degradation and then increases the levels of A3G both in cells and in newly-synthesized virions. In addition, because both MOV10 and A3G have anti-HIV-1 activity and belong to the interferon antiviral system, our findings suggested that these proteins are of synergistic anti-HIV-1 activities. These results will help us to get more comprehensive and profound understanding of MOV10 and A3G.

## Methods

### Plasmid construction and siRNAs synthesis

pcDNA3.1-A3G-HA, pcDNA3.1-GFP-HA, pcDNA3.1-MOV10-FLAG, pcDNA3.1-MOV10-HA, pcDNA3.1-rMOV10-FLAG, pcDNA3.1-MOV10-DEAG-mutant-HA, pcDNA3.1-MOV10-DEAG-mutant-FLAG and pcDNA3.1-Ub-FLAG were constructed as described previously [20, 32, 59]. pcDNA3.1-Vif-HA, pcDNA3.1-Vif-FLAG, pcDNA3.1-ElonginB-FLAG, pcDNA3.1-Cullin 5-FLAG, pcDNA3.1-CBF-β-FLAG, and were constructed by our lab [60]. HA or FLAG epitope tagged codon-optimized HIV-1 *vif* was constructed by chemically-synthesis of DNA fragment and subcloned into pcDNA3.1. Codon optimization was performed using the sequence of HIV-1_NL4-3_ [61]. FLAG epitope tag sequence at 3′ terminus of ElonginB, Cullin 5, or CBF-β was amplified through reverse transcription-polymerase chain reaction (RT-PCR) with the mRNA of 293T cells as the template. ElonginC with FLAG tag sequence at its 3′ terminus was amplified via PCR from the pElonginC-HA plasmid, which was generously provided by Dr. Xianghui Yu in Jilin University [62]. Then, the tagged ElonginB, ElonginC, Cullin 5, or CBF-β was inserted into pcDNA3.1 vector. HIV-1 proviral construct pNL4-3-ΔEnv-GFP has been described in our previous reports [63, 64]. pNL4-3-ΔEnv-GFP-ΔVif, a *vif* defective construct, was generated from pNL4-3-ΔEnv-GFP. The *vif* gene in pNL4-3-ΔEnv-GFP-ΔVif was disrupted by PCR-mediated site-directed mutagenesis and introduced nonsense mutations at codon positions 26, 27 (AAA, CAC → TAA, TAG) and/or 33, 34 (ACT, AAA → TAA, TAG) [65]. The vector pLKO.1-TRC, which contains a U6 promoter and *puromycin* selection gene and was obtained from Addgene (plasmid # 10878), was used for expression of MOV10-shRNA or scrambled control (Scr)-shRNA. Forward oligo of MOV10-shRNA (TRCN0000425452): 5′-CCGGGGCCAGTGTTTCGAGAGTTTCCTCGAGGAAACTCTCGAAACACTGGCCTTTTTG-3′; reverse oligo of MOV10-shRNA: 5′-AATTCAAAAAGGCCAGTGTTTCGAGAGTTTCCTCGAGGAAACTCTCGAAACACTGGCC-3′. Scr-shRNA forward oligo: 5′- CCGGAACGTACGCGGAATACTTCGACTCGAGTCGAAGTATTCCGCGTACGTTTTTTTG-3′; Scr-shRNA reverse oligo: 5′- AATTCAAAAAAACGTACGCGGAATACTTCGACTCGAGTCGAAGTATTCCGCGTACGTT-3′ [40]. All oligos were synthesized from Ribobio (Guangzhou, China).

The siGENOME SMART pool against MOV10 and siRNA for negative control were designed by Dharmacon and the target sequences for *MOV10*-specific siRNAs were chosen as described previously [20, 32]. *ElonginB*-specific siRNA, *ElonginC*-specific siRNA, and *Cullin 5*-specific siRNA were designed and synthesized by Ribobio (Guangzhou, China).

### Cell culture and transfection

Human 293T cells were obtained from American Type Culture Collection (ATCC) and grown at 37 °C with 5% CO2 in Dulbecco's modified Eagle's medium (DMEM) (Invitrogen) supplemented with 10% fetal bovine serum (FBS) (Invitrogen) and 1% penicillin–streptomycin (Invitrogen). The cells were transfected with the indicated plasmids or siRNAs by lipofectamine 2000 (Invitrogen). The procedures described by the manufacturer were followed.

### Co-immunoprecipitation and western blotting

Co-immunoprecipitation and western blotting assays were performed as previously described [20, 32]. In brief, human 293T cells were lysed with the lysis buffer (150 mM NaCl, 50 mM Tris–HCl [pH 7.5], 1 mM EDTA, 1% Triton X-100, 0.5% NP-40, plus PMSF and protease inhibitor cocktail [Sigma]) for 30 min at 4 °C. The cell lysates were clarified by centrifugation at 18,000*g* for 30 min at 4 °C, then mixed with anti-HA agarose beads (Sigma) and incubated at 4 °C for 4 h, followed by washing four times with cold lysis buffer and eluting in gel loading buffer. As indicated, the beads were treated with RNase mixture (DNase-free, Roche) (20 μg/ml) and incubated at 37 °C for 30 min. The immunoprecipitated samples were analyzed by SDS-PAGE and detected by western blotting. Anti-HA antibody (mouse monoclonal, Covance), anti-FLAG antibody (rabbit polyclonal, MBL), anti-GAPDH antibody (rabbit polyclonal, MBL), anti-MOV10 antibody (rabbit polyclonal, Abcam), anti-ElonginC antibody (rabbit polyclonal, Abcam), anti-Cullin 5 antibody (rabbit polyclonal, Abcam), anti-A3G antibody (rabbit polyclonal, Abcam), anti-Vif antibody (mouse monoclonal, Abcam), and anti-HIV-1 p24 antibody (rabbitpolyclonal antibodies made by our lab) were used as primary antibodies [64]. Quantity One program (Bio-rad) was used to quantify the western blotting results.

### HIV-1 virus-like particle (VLP) purification

Human 293T cells were transfected with pNL4-3-ΔEnv-GFP or pNL4-3-ΔEnv-GFP-ΔVif and other indicated plasmids. After 48 h of transfection, cell supernatants were collected, centrifuged at 4 °C for 10 min at 8000 rpm (≈ 7000 g) and filtered through a 0.45 μm filter to remove cellular debris. And the cell-free supernatants were concentrated by ultracentrifugation through a 20% sucrose cushions at 4 °C for 2 h at 45,000 rpm (≈ 40,000 g) (HITACHI Preparative Ultracentrifuge, CP80WX). Then, the pellets were re-suspended in RIPA buffer containing protease inhibitor cocktail and subjected to immunoblotting.

### Construction of MOV10-knockdown H9 cells

The pLKO.1-MOV10-shRNA or pLKO.1-Scr-shRNA was co-transfected with psPAX2 and pCMV-VSV-G into 293T cells. After 48 h, the supernatants were harvested and filtered with 0.45 μm filters (Millipore). Then, H9 cells were infected with MOV10-specific-shRNA-expressing or Scr-shRNA-expressing lentivirus respectively for 8 h and cultured with fresh medium. After 48 h, virus-infected H9 cells were selected by puromycin (1 μg/ml) for 2 weeks and subjected to the following experiments.

### Wild-type HIV-1 infection

MOV10-knockdown H9 cells and negative control cells were infected with HIV-1_NL4-3_ (p24 titer of 5 ng ml^−1^) for 3 h and then cultured with fresh medium and detected p24 in culture supernatant at different days. After 12 days, cells were collected and treated with the transcription factor buffer set including fixation/permeabilization and fixation/wash buffers (BD Biosciences) according to the manufacturer supernatant at different days. After 12 days, cellFITC-conjugated anti-HIV-1 p24 antibody (Santa Cruz Biotechnology) for intracellular HIV-1 Gag (p24) expression. Then, p24 positive cells were sorted with BD Aria sorter for further analysis. Data was analyzed with FlowJo software (Tree Star, Ashland, OR).

### Virus infectivity assay

Human 293T cells were co-transfected with pCMV-VSV-G, pNL4-3ΔEnv-GFP-ΔVif (or pNL4-3ΔEnv-GFP), and increasing amounts of pcDNA3.1-MOV10-FLAG or pcDNA31-MOV10-mutant-FLAG in the presence or absence of A3G-HA expressing plasmid. The virus-containing supernatant were collected at 48 h after transfection and filtered by a 0.45 μm filter. After normalization for HIV-1 p24 by enzyme-linked immunosorbent assay (ELISA, Clonetech), TZM-bl cells (2.5 × 10^5^ cells per well in 24-well plates) were infected with virus which containing 5 ng of p24 capsid. And then, luciferase enzyme activity determinations at 72 h post infection were carried out.

## Abbreviations

MOV10: Moloney leukemia virus 10
A3G: Apolipoprotein-B-mRNA-editing enzyme catalytic polypeptide-like 3G
USP: Ubiquitin–proteasome system
